# An Energy-Efficient Fall Detection Method Based on FD-DNN for Elderly People

**DOI:** 10.3390/s20154192

**Published:** 2020-07-28

**Authors:** Leyuan Liu, Yibin Hou, Jian He, Jonathan Lungu, Ruihai Dong

**Affiliations:** 1 Faculty of Information Technology, Beijing University of Technology, Beijing 100124, China; chinaleyuan@emails.bjut.edu.cn (L.L.); ybhou@bjut.edu.cn (Y.H.); Jtlungu77@hotmail.com (J.L.); 2 Beijing Engineering Research Center for IOT Software and Systems, Beijing University of Technology, Beijing 100124, China; 3 Insight Centre for Data Analytics, University College Dublin, Dublin 4, Ireland; ruihai.dong@ucd.ie

**Keywords:** fall detection, ZigBee, FD-DNN, energy-efficient

## Abstract

A fall detection module is an important component of community-based care for the elderly to reduce their health risk. It requires the accuracy of detections as well as maintains energy saving. In order to meet the above requirements, a sensing module-integrated energy-efficient sensor was developed which can sense and cache the data of human activity in sleep mode, and an interrupt-driven algorithm is proposed to transmit the data to a server integrated with ZigBee. Secondly, a deep neural network for fall detection (FD-DNN) running on the server is carefully designed to detect falls accurately. FD-DNN, which combines the convolutional neural networks (CNN) with long short-term memory (LSTM) algorithms, was tested on both with online and offline datasets. The experimental result shows that it takes advantage of CNN and LSTM, and achieved 99.17% fall detection accuracy, while its specificity and sensitivity are 99.94% and 94.09%, respectively. Meanwhile, it has the characteristics of low power consumption.

## 1. Introduction

According to the report issued by the World Health Organization (WHO), around 30% of people aged over 65 fall every year, and it leads to more than 300,000 people dying from falls each year [1]. Data from emergency department visitors were evaluated and identified; they indicated that 22.6% of the elderly fall victims suffered at least one new fall in six months [2]. Falling has been one of the leading causes of injuries to the elderly. It not only causes serious trauma to the brain, fractures, etc., but also causes psychological fear and brings great psychological trauma to the elderly [3]. If the elderly cannot be found and rescued in time when they fall, the fall will often cause serious injury or even death [4]. Therefore, the study of fall detection techniques is of great significance in reducing the mortality from falls in the elderly.

At present, the common fall detection systems [5] are as follows: the system with which the user actively initiates the alarm, the microphone-based fall detection system, the video-based fall detection system, and the fall detection system based on wearable sensors. For example, Cheffena used a support vector machine (SVM)-based method on collected microphone sounds to classify sounds of different activities [6]. The shortcomings of Cheffena’s research are that the fall detection needs to be in a quiet home environment, and the analysis of large amounts of audio data requires high energy consumption. Similarly, Harrou et al. adopted an SVM-based method to classify falls on video datasets, and achieved a high precision of 97.2% [7]. Debard et al. proposed a video detection method that was based on the static characteristics of the human body [8]. Although the video-based fall detection systems can achieve high accuracy, these systems often require a large amount of computing resources to process video data and are criticized for exposing user privacy. With the quick development of MEMS (microelectromechanical systems), the inertial sensor is becoming smaller and cheaper, and it has been widely used in wearable devices [9]. Researchers have developed a variety of fall detection technologies based on wearable devices [10,11,12]. For example, Qu et al. determined falls by analyzing the characteristics of the combined acceleration and differential acceleration of the sensor data [13,14]. The fall detection system developed by Qu et al. has a low accuracy of 90%. Salgado and Afonso introduced an extended Kalman filter to identify a real-time pose body model described by the accelerations from the tri-accelerometer sensor of a smartphone, and then an SVM performed a binary classification to detect falls [15]. Salgado and Afonso’s fall detection method has an accuracy of 96%, which is not accurate enough. The sensor-based mainstream fall detection technologies can be summarized into the following categories:

- The sensors collect human motion data and directly transmit them to the server for processing, and the server has powerful computing capabilities to run the fall detection algorithm. For example, Luo et al. designed a fall detection system that uses three-axis acceleration sensors to sense the acceleration data of the head, waist, and ankle in real time, and sends the data to the server in real time to detect the fall using a decision tree [16]. Since the module needs to continuously transmit data to the server, great energy consumption is the main shortage of this approach. In addition, the network will have a large delay when a large amount of data is to be transmitted.
- The fall detection algorithm runs on the wearable device without wireless transmission to the server. For example, S. Khojasteh et al. developed a wrist-worn sensory module, which was embedded with a three-axis accelerometer to collect wrist motion data. A decision tree and a rule-based algorithm were used to analyze the peaks of motion data for fall detection. Due to the limited computing power of wearable devices, only some simple fall detection algorithms can run on these devices [17]. As a result, the fall detection results are not accurate enough.
- Human motion data is pre-processed on the sensing device, and then a small amount of pre-processed data is wirelessly transmitted to the server for fall detection. For instance, Huynh et al. designed a cloud-based fall detection system that uses a threshold for fall detection. The information features of daily activities from sensor data are extracted and uploaded to a cloud server periodically for later analysis and daily activities classification [18]. This method can not only save the energy of the wearable device, but also use a high-precision algorithm to improve the fall detection accuracy. Hence, there are many studies using this method. However, there are still many problems to be solved with this method, including simplifying the pre-processing algorithm and improving battery life.

To solve the problems of high energy consumption and insufficient accuracy of the fall detection technology, this paper introduces a novel wearable module to sense motion data by using the low-power chips and the MUP6050 sensor integrated with the three-axial accelerometer and the three-axial gyroscope. Based on the interrupt function of MPU6050, a low-power algorithm is developed to control the module’s data sensing process and the data transmission process. Furthermore, the six-dimensional (3D acceleration and 3D angular velocity) data are received through a sliding window at the receiving end (namely, the server), and a deep neural network (FD-DNN) based on a convolutional neural network (CNN) and long short-term memory (LSTM) for fall detection is designed to run on the server, so as to distinguish between falls and activities of daily life (ADL). The paper is structured as follows: The methodology of sensing and transmitting human motion data with low power consumption is introduced in Section 2. The architecture of FD-DNN and the way to train and implement FD-DNN are presented in Section 3. Section 4 describes the experimental process and analyzes the experimental results. Section 5 summarizes the full text and introduces future research directions.

## 2. Sensing Motion Data

The module of sensing human motion data is firstly introduced, and then an interrupt-driven control algorithm of the module is introduced. Based on the purpose of low power consumption, a variety of low-power chips and sensors are selected comprehensively, combined with a control algorithm to achieve low power consumption of the fall detection module.

### 2.1. Energy-Efficient Data Sensing Module

**Figure 1:**
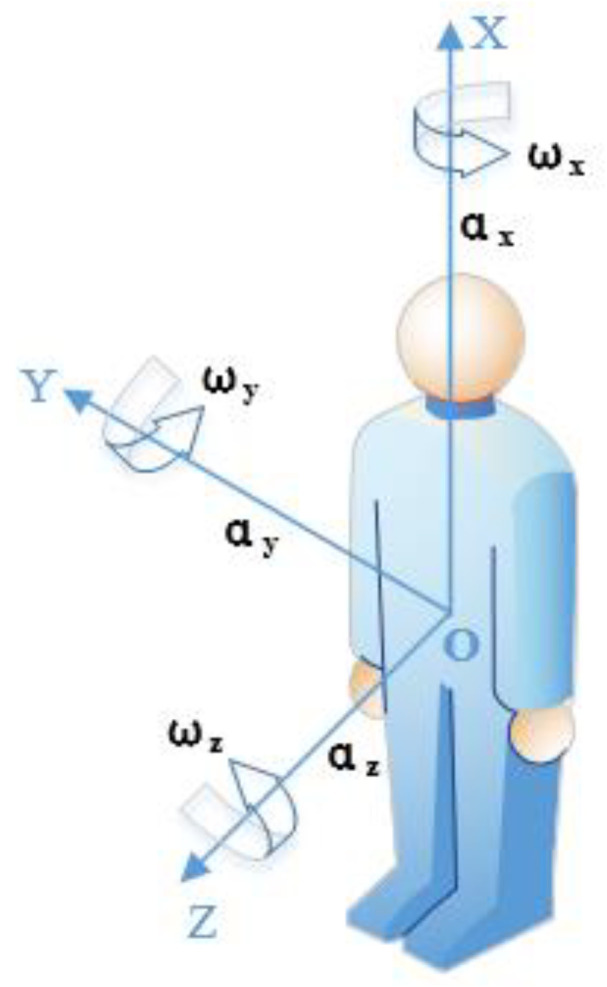
The model of human motion.

According to the research results from Gia et al. [19], the area above the human waist has a small range of motion, which is more suitable to collect acceleration and angular velocity data so as to distinguish falls from ADLs. Taking system reliability and wearing comfort into account, we place the motion sensing module at the waist position through a specific vest and model the human motion according to the placement position in a Cartesian coordinate system [20]. Figure 1 shows the human motion model, in which *α_x_*, *α_y_*, and *α_z_* are accelerations along the *X*, *Y*, and *Z* axes, respectively, and *ω_x_*, *ω_y_*, and *ω_z_* are angular velocities along the *X*, *Y*, and Z axes, respectively.

**Figure 2:**
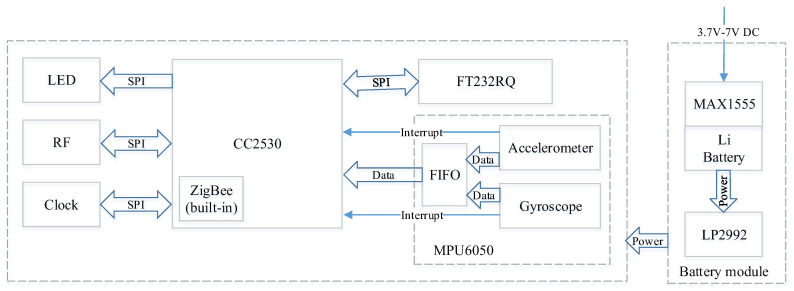
The module layout.

Based on the above model of human motion, we designed a module to sense human motion data, and Figure 2 shows its layout. The module uses the MPU6050 integrating with a three-axial MEMS gyroscope and accelerometer to capture the acceleration and angular velocity data. The acceleration range that MPU6050 can measure is −16 g to + 16 g, and the angular velocity range that it can measure is −2000 degrees per second to +2000 degrees per second. The CC2530 from Texas Instruments is used as the central processing unit, and its frequency is 16 MHz. By controlling the sleep and wake-up states of CC2530, energy consumption can be saved. The low-power ZigBee chip built in the CC2530 unit is used as the transceiver. Its maximum transmitting distance is about 100 m, and its data transmission rate is up to 250 kb/s. According to Bet et al. [21], the sensors used in most fall detection studies have sampling frequencies between 40 Hz and 200 Hz. To ensure that high-frequency components are not discarded and the amount of data generated is not too large, the sampling frequency of the designed sensing module is set to 100 Hz. In addition, the LP2992 lithium battery control chip, the MAX1555 charge controller, the LP2992 micro-power voltage stabilization chip, and the FT232RQ serial port management chip are selected to form the data sensing module. Low-power microcontrollers, low-power ZigBee, MEMS sensors, cache, and micro-power power management were comprehensively integrated to achieve the low-power goal of the data sensing module.

### 2.2. Energy-Efficient Data Sensing and Transmission Algorithm

According to the instructions of the MPU6050 [22], the free fall interrupt will be triggered when the absolute values of all three accelerometer axes are less than the prescribed threshold in the FF_THR register and the duration of the free fall reaches the prescribed threshold in the FF_DUR register. The motion interrupt will be triggered when the absolute value of any accelerometer axis exceeds the prescribed threshold in the MOT_THR register and the duration reaches the prescribed threshold in the MOT_DUR register. The zero motion interrupt will be triggered when the absolute values of all three accelerometer axes are less than the prescribed threshold in the ZRMOT_THR register and the duration reaches the prescribed threshold in the ZRMOT_DUR register.

When the human body falls, there will be a period of weightlessness, and the acceleration will quickly decrease to 0, which will trigger the free fall interrupt of the MPU6050. When the angle between the direction of gravity and the three axes of the accelerometer are the same, the maximum value of the component of gravity on the three axes reaches a minimum of 0.57735 g. If the threshold is set greater than 0.57735 g and the accelerometer is placed in the special way, it will always be misjudged as the free fall interrupt. Thus, the FF_THR is set to 0.563 g. Because weight loss during human fall lasts less than 20 ms according to reference [18], the FF_DUR is set to 20 ms. Hence, when a free fall interrupt is captured by the system and it lasts longer than 20 ms, the CC2530 will be activated to control the ZigBee to transmit the data in the FIFO buffer to the server, in order that the judgment could be made by the fall detection algorithm. When the accelerometer is placed at rest and placed in the special way, the acceleration on the three axes of the accelerometer is 0.57735 g. The larger the threshold setting of the ZRMOT_THR, the looser the requirements for triggering the zero motion interrupt. In order to make the accelerometer sensitive enough to detect zero motion and distinguish it from free fall, the ZRMOT_THR is set to 0.5 g and the ZRMOT_DUR to 1000 ms. Namely, when the acceleration sensor’s value is less than 0.5 g and the delay is more than 1 s, a zero motion interrupt will be triggered and the module will run into sleep mode. In this case, acceleration and gyroscope data are sampled and stored into the FIFO through by the MPU6050 in low-power mode. In addition, the data sampling frequency of the sensor module is set to 100 Hz, and each piece of data produced by the MPU6050 sensor is 16 bits, occupying 2 bytes, so the FIFO_CNT is set to 600 bytes to store 0.5 s of data from the accelerometer and gyroscope. As a result, when the data stored in the FIFO reaches 600 bytes, the FIFO interrupt will be triggered.

**Figure 3:**
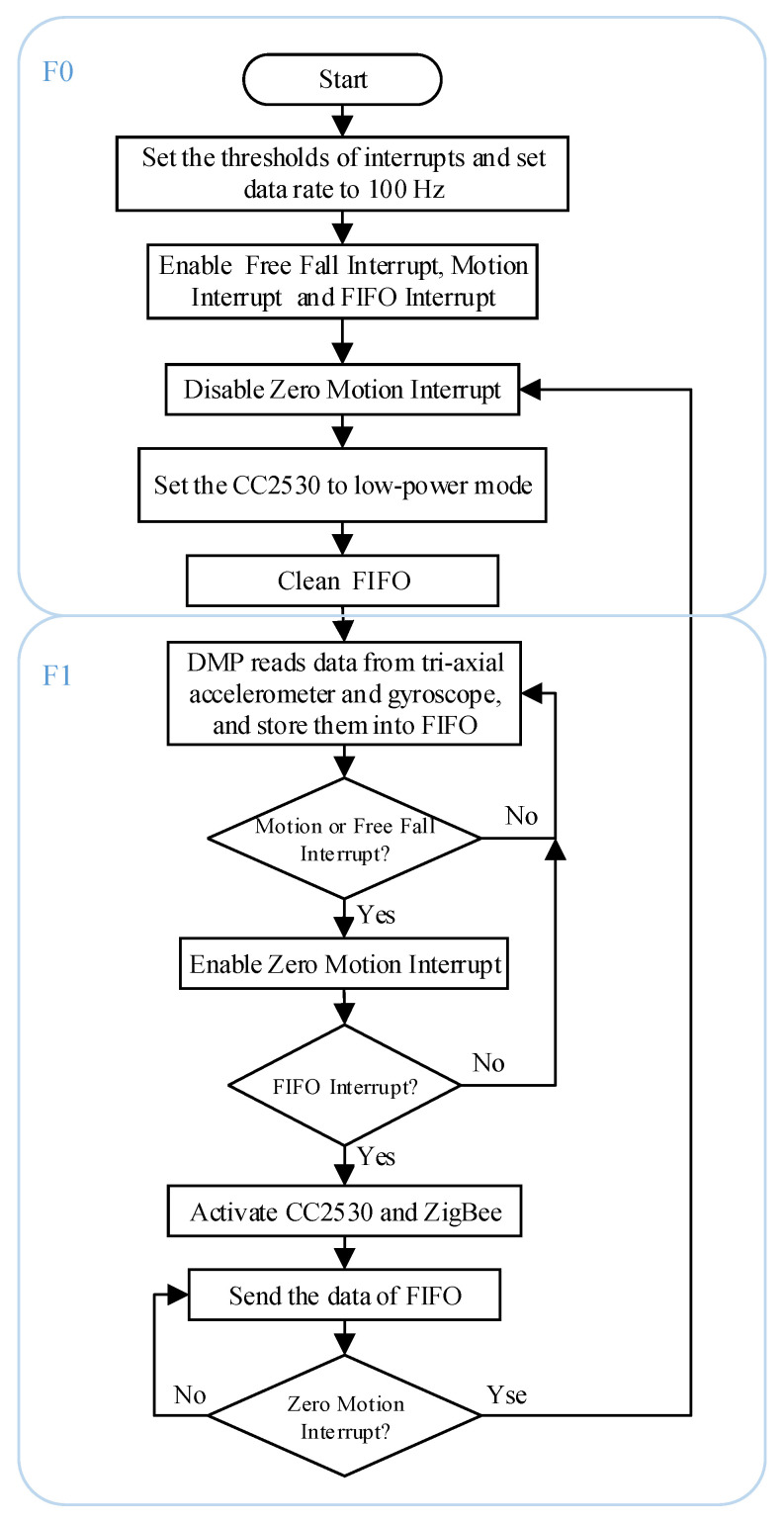
The flowchart of the data sensing and transmission algorithm.

Based on the MPU6050 interrupt system, the data sensing algorithm is designed, and Figure 3 shows its flowchart. A finite state machine is used to model the algorithm. The state machine has 3 states. The tasks and the relationships between the states are as follows:

F0: initial state. The thresholds of FF_THR, FF_DUR, MOT_THR, MOT_DUR, ZRMOT_THR, ZRMOT_DUR, and FIFO_CNT are configured. The free fall interrupt, the motion interrupt, and the FIFO interrupt are enabled and the zero motion interrupt is disabled. The data rate of MPU6050 is set to 100 Hz. At last, the CC2530 enters low-power mode and the state machine enters F1.

F1: motion state. The DMP reads data from the three-axial accelerometer and gyroscope and stores them into FIFO. When the data in the FIFO are full, the FIFO interrupt will be triggered and the data will be updated according to the FIFO principle. When the free fall interrupt or motion interrupt is triggered, the CC2530 will be activated to control the ZigBee to transmit the data in the FIFO buffer to the server, and the zero motion interrupt will be enabled. If the FIFO interrupt is not captured by the system, the data in FIFO will be continuously updated based on the FIFO principle. Else, if the FIFO interrupt is captured, the data in the FIFO buffer will be continuously transmitted to the server until a zero motion interrupt is triggered. After the zero motion interrupt is triggered, the state machine enters F0.

Through the data sensing and transmission algorithm, the sleep and wake-up modes of CC2530 and ZigBee can be controlled, and the conditional transmission of data can be realized instead of constant transmission. Therefore, the low-power scheduling algorithm in this section and the low-power hardware in Section 2.1 jointly realize the system-level low-power consumption of the system.

## 3. Deep Neural Network for Fall Detection

Since the convolutional layer of CNNs can extract features from the input data that cannot be acquired manually [23], and the LSTM is suitable for processing important events with temporal dynamics [24], we developed a novel framework (FD-DNN) for fall detection which combines convolutional and recurrent layers. The convolutional layers extract features and provide abstract representations (feature maps) of the human activity data. The recurrent layers model the temporal dynamics of the activation of the feature maps.

**Figure 4:**
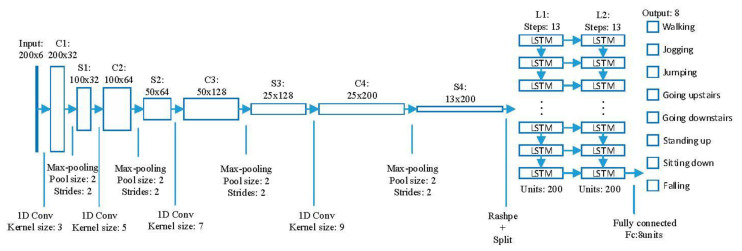
The architecture of the FD-DNN.

The architecture of the FD-DNN model is shown in Figure 4. It consists of one input layer, four convolution layers, four max-pooling layers, two LSTM layers, one fully connected layer, and one output layer, which are labeled as Input, Conv, Max-pooling, LSTM, Dense, and Output.

Since the duration of a human fall often does not exceed two seconds, our application uses a two-second sliding window to process the received six-dimensional (3D acceleration and 3D angular velocity) data, the rate of which is 100 Hz. Namely, the sliding window contains totally 200 sets of six-dimensional data. Hence, the input of the network is 200 sets of six-dimensional sensing data.

The first layer (C1) is a 1D convolutional layer with 32 filters, each 3 × 6, using a stride of 1. The filters are used to extract the features in the sensing data. The required filters are obtained by self-learning parameters of the filters during FD-DNN training. When a certain filter filters the input data matrix, the filter slides along the length of the input matrix. Each time the filter slides to a position, the filter performs a dot product on the input matrix area of the same size as the filter, and then sums the values of the dot products to obtain the filtered eigenvalue. The SAME padding is used for edge padding to avoid information loss. The filter filters all regions along the length of the input matrix to generate a 200 × 1 matrix. Each of the outputs is activated by a rectified linear unit (ReLU). Since C1 has 32 filters, layer C1 generates a 200 × 32 output matrix, and it contains 608 (32 × 3 × 6 + 32) trainable parameters.

The following layer (S1) is a max-pooling layer, which uses a pool size of 2 × 1 with a stride of 2 × 1 and the SAME padding. It reduces the data in half in the temporal dimension. The max-pooling layer has no trainable parameter [25].

Next, the similar structure (a 1D convolutional layer followed by a max-pooling layer) is repeated three times: the convolutional layer (C2) with 64 filters, each 5 × 32, using a stride of 1 is followed by the max-pooling layer (S2); the 1D convolutional layer (C3) with 128 filters, each 7 × 64, is followed by the max-pooling layer (S3); and the 1D convolutional layer (C4) with 200 filters, each 9 × 128, is followed by the max-pooling layer (S4). The output shape of S4 is (batch, 13, 200), which is reshaped and split into 13 (batch, 200) matrices as the input of the next layer (L1).

Layers L1 and L2 are two LSTM layers; each has 200 units. Karpathy et al. proved by experiment that the depth of at least two recurrent layers is beneficial when processing sequential data [26], so the network is set up as two LSTM layers. Overfitting often occurs during neural network training, resulting in models that usually do not achieve the best performance. The specific performance of overfitting is that the model has a small loss function on the training data and a high prediction accuracy, but the model has a large loss function on the test data and the prediction accuracy is low. When training neural networks, overfitting is a frequent problem. Therefore, a dropout mechanism [27] has been added to the cells of each LSTM layer, in order to prevent overfitting and improve performance. Dropout makes each LSTM cell work normally with a certain probability when data flows in; otherwise, the LSTM cell outputs a value of 0. The tanh (hyperbolic tangent) function is used as the activation function of recurrent units. The reason for using tanh function is that its second derivative of the hyperbolic tangent function can sustain for a longer range before going to zero, which can overcome the disappearing gradient problem. At the same time, the LSTM layer with tanh as the activation function has the advantages of faster convergence and simpler gradient calculation in practice. L1 and L2 share 320,800 trainable parameters. The output of the last cell of L2 will be used as the input of the next layer (Fc).

Fc is a fully connected layer with 8 units (one for each label “walking”, “jumping”, etc.). Each neuron in the fully connected layer is connected to all neurons in the previous layer to synthesize the extracted features. Softmax shown in Formula (1) was added to the Fc layer to calculate the probability of each class. The softmax function shown in Equation (1) plays the role of classifier. The calculation result $s_{i}$ of Formula (1) is the probability of each class. The sum of the probabilities of all classes is 1. The class with the highest probability will be selected as the prediction result. $x_{1},x_{2}\ldots x_{j}$ are the original inputs of the full connection layer, and n is the number of categories of the classification.

(1) $$softmax\left(x_{i}\right)=s_{i}=\frac{e^{x_{i}}}{\sum_{j=1}^{n}e^{x_{j}}}$$

## 4. Experiments and Evaluation

This section describes the setup of the experimental environment, the construction of the dataset (including collection from the experimental environment), model training methods, results, and discussion.

### 4.1. The Experimental Environment

**Figure 5:**
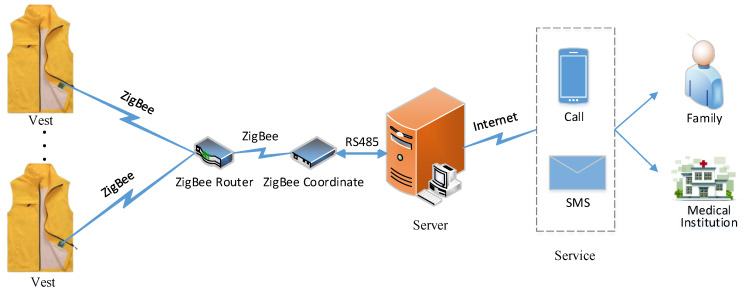
The experimental environment for fall detection.

The experimental environment shown in Figure 5 was used for fall detection experiments, and the configuration of the serve is shown in Table 1. The MUP6050 and Zigbee-integrated sensing module was fixed to the front waist of the wearable vest. The sensing module collected the data of human activities and stored them in the FIFO or sent them to the server according to the data sensing algorithm described in the second section. The server appended the received data to the sliding window, which was about two seconds long and followed the FIFO principle. The data were normalized by Formula (2) on the server and then filtered by the moving average filter. Meanwhile, the FD-DNN was utilized to classify the data in the sliding window. The system will automatically alert the guardian by phone, or by sending text messages with the GPS location as soon as a fall is detected.

The data of daily activities—including walking, jumping, jogging, going upstairs, going downstairs, standing up, sitting down, and falling—were collected by the experimental environment shown in Figure 5. Motion data for each activity were collected to build the dataset, which will be described in detail in the next section.

### 4.2. Datasets

As the basis for training and testing our FD-DNN model, we construct a new Joint-Dataset. Firstly, we cleaned up and extracted some data from two open datasets—SisFall [28] and MobiFall [29], respectively. Furthermore, through our well-designed experimental environment described in the previous section, we collected 1600 extra samples for eight different activities.

**Figure 6:**
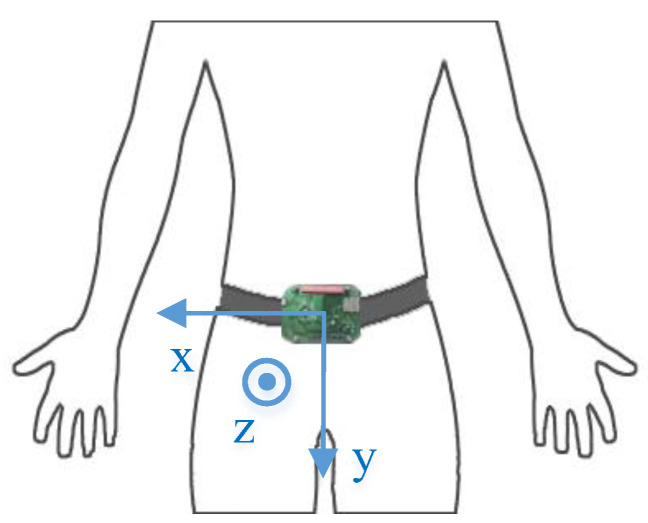
The SisFall data sensing device’s position and coordinate system.

The SisFall dataset includes 19 types of ADLs and 15 types of falls. The experiment was performed by 38 adults, among whom 14 healthy and independent elderly subjects over 61 years old only performed 15 types of ADLs, and one elderly subject of 60 years old and 23 young subjects aged from 19 to 30 performed both ADLs and falls. The dataset was recorded with a self-developed device integrated with a three-axial accelerometer and a gyroscope. During the experiment, the device was fixed to the waist of a subject, which is demonstrated in Figure 6. It can be seen that its coordinate is slightly different from the human motion model.

The MobiFall dataset directly collected the motion data of three-axial acceleration and angular velocity from a Samsung Galaxy smartphone, which was placed in the subject’s trouser pocket in a random orientation. The MobiFall dataset also included the orientation data about the subject’s smartphone. Twenty-four volunteers (22 to 42 years old) performed nine types of ADLs and four types of falls. Among them, nine subjects performed both falls and ADLs, and 15 other subjects only performed falls.

First, the coordinates of the SisFall dataset and the MobiFall dataset are transformed into the coordinate system shown in Figure 1. In addition, the sampling frequency of both datasets is 200 Hz, while the sampling frequency proposed in this paper is 100 Hz. Hence, both SisFall and MobiFall datasets were downsampled to 100 Hz. Taking the different ranges and precision of the sensors used in the SisFall and MobiFall datasets into account, the two datasets were normalized by Formula (2) according to the range specification of each sensor.

(2) yi=xi−{xj}1≤j≤nmin{xj}1≤j≤nmax−{xj}1≤j≤nmin

Since the noises among the motion data always have significant effect on fall detection accuracy, it is necessary to filter the noises [30]. Xiao et al. [31] used the mean filter, the moving average filter, and the Prewitt horizontal edge-emphasizing filter to filter noise in the swimming recognition. The experiments proved that the moving average filter has the best effect on filtering noise. Therefore, the moving average filter depicted in Formula (3) is introduced to filter noise. In Formula (3), *G* is the original data, *M* is the size of the sliding window, and $G_{filter}$ is the filtered data.

(3) $$G_{filter}\left(n\right)=\frac{1}{M}\left(G\left(n\right)+G\left(n-1\right)+\cdots +G\left(n-M+1\right)\right)$$

After filtering the noise, we extracted 1000 samples for each activity from the SisFall dataset and the MobiFall dataset. Among them, the samples of walking, jumping, jogging, going upstairs, and going downstairs were extracted from the MobiFall dataset. The samples of standing up and sitting down were extracted from the SisFall dataset. The samples of falling consisted of 500 copies from MobiFall and 500 copies from SisFall. Overall, there were 8000 samples. Among them, each kind of ADLs and falls had 1000 samples, respectively.

Furthermore, the experimental environment in Section 4.1 was used to collect some motion data as part of the Joint-Dataset. Twenty volunteers aged 24 to 50 participated in the data collection. 17 of them were male and three were female. There were eight activities, including walking, jogging, jumping, going upstairs, going downstairs, standing up, sitting down and falling. Each volunteer repeated each activity 10 times, so 1600 data samples were collected.

The 1600 sets of data collected by the experiment were combined with the 8000 sets of data extracted from the public datasets into a new dataset called the Joint-Dataset. The composition of Joint-Dataset is shown in Table 2. The Joint-Dataset was used for network training and testing. 80% of the Joint-Dataset was used for training, and the remaining 20% was used for testing. Table 3 shows the training data, validation data, and test data from the Joint-Dataset.

### 4.3. Methodology

The FD-DNN was implemented with the TensorFlow framework. The training and classification of the model were carried out using the server shown in Figure 5. Data were read and processed using the Pandas and Numpy libraries. Before training, the activity tags were one-hot encoded to simplify the logic and speed up the network calculation. The model was trained in a fully-supervised way, back propagating the gradients from the fully connected layer to the convolutional layers.

The FD-DNN network was trained by the training dataset shown in Table 3. During the training, data were segmented into mini-batches of a size of 128 data segments, and the Adam algorithm was used to update the weights of the network. Since there were 200 data per sample, the step size of the network was set to 200. The learning rate was set to 0.001 and the number of epochs was set to 300.

In the convolutional layers and the max-pooling layers, the parameters such as the number of filters, the size of the convolution kernel, the sliding stride, and the edge padding method were set according to Section 3. In the two LSTM layers, the biases of the forget gate was set to 1.0 in order to reduce the scale of forgetting in the beginning of the training. The cell state and the hidden state of each LSTM layer were all initialized to zero. A dropout mechanism was added to each LSTM layer, and the activation of each randomly selected unit of each LSTM layer was set to zero with a probability of 0.5. In the full connection layer, the loss was calculated according to Formula (4). Based on the loss, the parameters were optimized.

(4) $$loss=-\frac{1}{n}\sum_{i=1}^{n}y_{i}^{\text{′}}\log\left(y_{i}\right)$$

The Adam optimizer, which combines the ability of AdaGrad [32] to handle sparse gradients and the ability of RMSProp [33] to handle non-stationary targets, was selected to optimize the network parameters [34]; it calculated the adaptive learning rates for different parameters based on the estimates of the first and second moments of the gradient. For the sake of effect, the parameters were optimized with a learning rate of 1 × 10^−3^ and a numerical stability variable of 1 × 10^−8^. The parameters were updated according to the Adam method after each mini-batch.

Based on the above settings, the training was carried out. Training loss and training accuracy were computed every five iterations. Validation loss and validation accuracy were computed at every 25 iterations. The trained model is saved every 10 epochs. At the beginning of training, the accuracy of the FD-DNN network improved very quickly and fluctuated greatly. At the end of training, the accuracy improved slowly and fluctuated slightly. After 136-epochs training, the network accuracy (tested by the verification dataset) reached 99.28%, and no longer improves when training continues. Thus, the network training was stopped and the FD-DNN network model was saved. Since the FD-DNN was implemented using the TensorFlow framework, the saved model is a file with the suffix ‘.ckpt’, which can be used for various tests.

After training, the trained and saved FD-DNN model was used for offline tests. First, the test data were read and the trained model was loaded. Then, the test data were input to the loaded model. Finally, the test loss, test accuracy, and confusion matrix of the test are output. In addition, the specificity and sensitivity were calculated according to the confusion matrix. The test results were combined with the verification results during training to evaluate the performance of the network.

### 4.4. Results and Discussion

The test dataset was used to test the trained FD-DNN network offline. After testing, the accuracy of FD-DNN was 99.17%, and the sensitivity and specificity were 94.09% and 99.94%, respectively. In addition, the CNN and LSTN algorithms were also used to do an offline experiment with the same training and test samples. Table 4 shows the comparison of accuracy, sensitivity, and time consumption. As shown in Table 4, the accuracy of the FD-DNN is about 1% higher than that of the CNN and about 2.3% higher than that of LSTM, the sensitivity is increased by about 7%, and the specificity is also improved. In addition, it only takes about 1.05 s to classify 1920 samples. It proves that FD-DNN takes advantages of CNN and LSTM.

Comparative experiments between FD-DNN and traditional classification algorithms were also performed. The Weka software integrates many traditional algorithms [35], so the dataset shown in Table 3 was imported into Weka to test the performance of different algorithms. Table 5 shows the experimental result. Among the traditional classification algorithms in Table 5, SimpleLogistic algorithm got the lowest accuracy, while it took about 0.19 s to test the model on testing data. The accuracy of FD-DNN is 19.44% higher than that of the SimpleLogistic algorithm, although the test speed of FD-DNN is 0.86 s slower than that of the SimpleLogistic algorithm. The Random Forest algorithm got the high accuracy, while it took about 1.57 s to test model on the test dataset. The accuracy and test time of FD-DNN are 4.67% higher and 0.52 s shorter than the Random Forest algorithm, respectively. The specificity of FD-DNN is higher than the other algorithms except Bayes Net, although the sensitivity is lower than the other algorithms except the Random Tree algorithm. While ensuring specificity and testing speed, the FD-DNN improved the accuracy significantly compared with the traditional classification algorithms.

The confusion matrix on the test dataset for the fall detection task is illustrated in Table 6 for the FD-DNN approach. According to the data in Table 6, the accuracy, sensitivity, and specificity of the fall detection can be calculated. It can be seen from Table 6 that the FD-DNN tends to misclassify Sitting down and Standing up. In the case of activity Sitting down, four Sitting down samples were misclassified as Standing up. Similarly, there were six Standing up samples that were misclassified as Sitting down. It may be because the misclassified samples of Sitting down and Standing up are not standard, resulting in the similarity of the two kinds of misclassified samples.

10 students aged 22–30 were invited to participate in online experiments. They wore the vest and repeated each of the eight activities 10 times. The server real-time received and classified the data sent from the sensor board in the vest. The accuracy of the online fall classification reached 98.25%, while the sensitivity and specificity were 87.72% and 100.00%, respectively. This shows that the proposed algorithm and system have good performance and can meet the requirements of real-time fall detection.

In order to verify the fall detection effect of the system for people over 65 years old, eight volunteers over 65 years old were invited to participate in the experiment. Their information is shown in Table 7. Each subject tried 10 times for each activity, so a total of 640 samples were collected. To the best of our knowledge, there are no studies to conduct fall experiments for elderly people over 65 years old due to the relatively high risk. Some emergencies also appeared during the experiments. Subject 1 suddenly had difficulty breathing during the experiments, which was relieved after taking the drug. Subject 3 sweated too much during the experiments, and the experiments had to be suspended several times. In spite of the emergencies, the volunteers persisted in completing the experiment.

The elderly data (the data of the subjects over the age of 65) were input into the previously trained FD-DNN model for testing, and the accuracy was only about 20%. The reason of low accuracy is that the motion of the elderly is slower and smaller than that of the young, and the data features are inconsistent with the features that can be recognized by the FD-DNN model trained on the young subjects’ data.

In order to enable the model to recognize the motion data of the elderly, 3/4 of the elderly data were mixed with the Joint-Dataset to retrain the model. After 140-epochs training, the accuracy and loss of FD-DNN no longer changed significantly, and the training was stopped. The test accuracy of the model was 99.22%, the specificity was 99.88%, and the sensitivity was 94.82%. In addition, the remaining 1/4 of the elderly data was used to test the newly trained model. The accuracy, specificity, and sensitivity of the test were 94.38%, 100%, and 81.63%, respectively. Too few elderly data is the reason for the low accuracy. If there are more elderly data to train the model, the test accuracy will be further improved.

**Figure 7:**
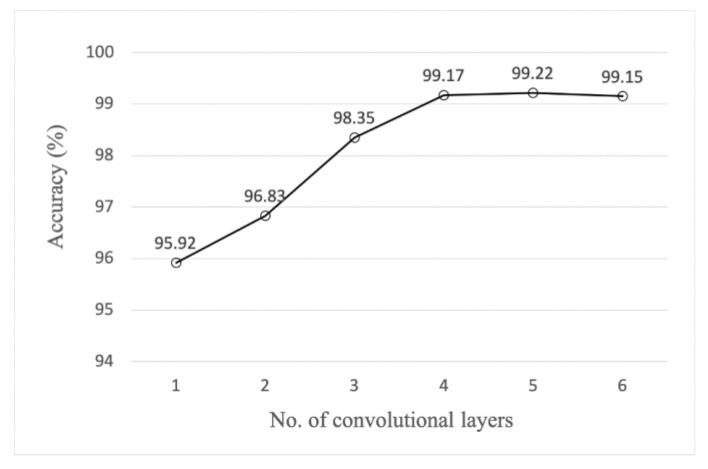
Ablation test accuracy of different convolutional layers.

Other research showed that increasing the number of convolutional layers may be beneficial for activity recognition [36]. Experiments were performed on the proposed FD-DNN with different number of convolutional layers. As can be seen from Figure 7, the accuracy has a large increase when the number of convolution layers grows from one to four, and the growth rate is smaller when the number of convolution layers is greater than four. In particular, the accuracy of six convolution layers is a little smaller than that of five convolution layers. Furthermore, the growing number of convolutional layers leads to a sharp increase of trainable parameters and computational resources consumption. For example, adding a C5 layer the same as C4 will increase the parameters by 460,000 and increase the RAM consumption by 304 M when training. Therefore, the number of convolutional layers in FD-DNN is set to four.

Two experiments were designed to verify the energy saving of the sensing module. A 600 mAH battery was used to power the sensing module, and an experiment was designed to make the sensing module continuously send the data in the cache to the server every 0.8 s. The experimental results showed that the sensing module can work in this way for more than 30 h. In another real-world experiment, two experimenters wore the vests embedded with the sensing module (powered by a 600 mAH battery) to work normally in the laboratory for eight hours a day. The experimental results showed that the sensing module can work continuously for 140 h on a full charge. The power experiments show that the interrupt-driven, ZigBee-based activity sensing module has the characteristics of low power consumption and can meet the needs of elderly people to detect falls.

## 5. Conclusions

This paper presents a novel low-power fall detection workflow for the elderly. Firstly, an interrupt-driven low-power motion sensing module was designed to sense and transmit the data of human activities. Secondly, the server-side FD-DNN was carefully designed to distinguish between falls and ADLs. Finally, experiments were carried out both on the offline dataset and online datasets. The experimental results show that the FD-DNN can effectively distinguish falls from daily activities. The FD-DNN takes advantages of CNN and LSTM, which achieved higher accuracy of fall detection than those of traditional classification algorithms. In addition, the method introduced in this paper can achieve the battery life of more than 140 h in real life. Besides, its low-power feature is practically available for fall detection in the elderly. The future work will focus on the ways to simplify the network and improve the speed for fall detection.

## Figures and Tables

**Table 1:** Platform configuration of the server.

Platform	Configuration
Operating system	Ubuntu 16.04.1 LTS
CPU	Intel(R) Xeon(R) CPU E5-2620 v4 @ 2.10 GHz
GPU	NVIDIA Tesla M40 12 G
RAM	125 G
Data processing	Pandas, Numpy
Deep learning framework	TensorFlow 1.3.0

**Table 2:** The composition of the Joint-Dataset.

Activity	Joint-Dataset		
	Samples from SisFall Dataset	Samples from MobiFall Dataset	Samples Collected by the Designed Module
Walking	0	1000	200
Jogging	0	1000	200
Jumping	0	1000	200
Going upstairs	0	1000	200
Going downstairs	0	1000	200
Standing up	1000	0	200
Sitting down	1000	0	200
Fall	500	500	200
Total	2500	5500	1600

**Table 3:** The Joint-Dataset for experiment.

Activity	Training Data	Validation Data	Test Data
Walking	840	120	240
Jogging	840	120	240
Jumping	840	120	240
Going upstairs	840	120	240
Going downstairs	840	120	240
Standing up	840	120	240
Sitting down	840	120	240
Fall	840	120	240
Total	6720	960	1920

**Table 4:** Performance comparison on FD-DNN, CNN, and LSTM.

Algorithm	Accuracy (%)	Sensitivity (%)	Specificity (%)	Test Time(S)
FD-DNN	99.17	94.09	99.94	1.05
LSTM	96.88	81.47	99.57	3.87
CNN	98.13	87.50	99.88	0.65

**Table 5:** Performance comparison on different algorithms.

Algorithm	Accuracy (%)	Sensitivity (%)	Specificity (%)	Test Time (S)
FD-DNN	99.17	94.09	99.94	1.05
Naive Bayes	90.10	95.65	99.93	8.87
Bayes Net	94.07	97.58	100.00	3.98
Random Forest	94.50	99.03	99.93	1.57
Random Tree	80.32	92.27	98.51	1.21
Bagging	91.29	97.58	99.73	0.04
J48	84.65	95.65	99.26	1.12
LogitBoost	81.80	99.65	99.80	0.05
SimpleLogistic	79.73	98.07	99.80	0.19

**Table 6:** Confusion matrix of offline test.

		Predicted Activity							
		Falling	Standing Up	Walking	Jogging	Jumping	Going Upstairs	Going Downstairs	Sitting Down
Actual Activity	Falling	239	0	0	0	0	0	0	1
	Standing up	0	234	0	0	0	0	0	6
	Walking	0	0	240	0	0	0	0	0
	Jogging	0	0	0	239	0	1	0	0
	Jumping	0	0	0	0	240	0	0	0
	Going upstairs	0	0	0	0	0	238	2	0
	Going downstairs	0	0	0	0	0	2	238	0
	Sitting down	0	4	0	0	0	0	0	236

**Table 7:** Information for volunteers over 65.

ID	Age	Gender
1	65	male
2	65	female
3	66	male
4	67	male
5	68	female
6	68	male
7	69	female
8	69	female
